# Electrochemical Immunosensors Developed for Amyloid-Beta and Tau Proteins, Leading Biomarkers of Alzheimer’s Disease

**DOI:** 10.3390/bios13070742

**Published:** 2023-07-17

**Authors:** Abhinav Sharma, Lúcio Angnes, Naghmeh Sattarahmady, Masoud Negahdary, Hossein Heli

**Affiliations:** 1 Solar Center, Physical Sciences and Engineering Division, King Abdullah University of Science and Technology (KAUST), Thuwal 23955-6900, Saudi Arabia; 2 Department of Fundamental Chemistry, Institute of Chemistry, University of São Paulo, Av. Prof. Lineu Prestes, 748, São Paulo 05508-000, Brazil; 3 Department of Medical Physics, School of Medicine, Shiraz University of Medical Sciences, Shiraz, Iran; 4 Nanomedicine and Nanobiology Research Center, Shiraz University of Medical Sciences, Shiraz, Iran

**Keywords:** electrochemical immunosensors, Alzheimer’s disease (AD), amyloid-beta (Aβ), tau proteins, molecular diagnostics

## Abstract

Alzheimer’s disease (AD) is the most common neurological disease and a serious cause of dementia, which constitutes a threat to human health. The clinical evidence has found that extracellular amyloid-beta peptides (Aβ), phosphorylated tau (p-tau), and intracellular tau proteins, which are derived from the amyloid precursor protein (APP), are the leading biomarkers for accurate and early diagnosis of AD due to their central role in disease pathology, their correlation with disease progression, their diagnostic value, and their implications for therapeutic interventions. Their detection and monitoring contribute significantly to understanding AD and advancing clinical care. Available diagnostic techniques, including magnetic resonance imaging (MRI) and positron emission tomography (PET), are mainly used to validate AD diagnosis. However, these methods are expensive, yield results that are difficult to interpret, and have common side effects such as headaches, nausea, and vomiting. Therefore, researchers have focused on developing cost-effective, portable, and point-of-care alternative diagnostic devices to detect specific biomarkers in cerebrospinal fluid (CSF) and other biofluids. In this review, we summarized the recent progress in developing electrochemical immunosensors for detecting AD biomarkers (Aβ and p-tau protein) and their subtypes (AβO, Aβ_(1-40)_, Aβ_(1-42)_, t-tau, cleaved-tau (c-tau), p-tau_181_, p-tau_231_, p-tau_381_, and p-tau_441_). We also evaluated the key characteristics and electrochemical performance of developed immunosensing platforms, including signal interfaces, nanomaterials or other signal amplifiers, biofunctionalization methods, and even primary electrochemical sensing performances (i.e., sensitivity, linear detection range, the limit of detection (LOD), and clinical application).

## 1. Introduction

Alzheimer’s disease (AD) is a chronic and progressive neurodegenerative disease that is becoming a serious global health issue. The disease leads to memory loss, personality changes, dementia, and more severe causes of death in elderly people [1,2,3]. The most common feature of AD is dementia, which may account for 60–70% of AD cases [4]. Today, dementia is one of the leading causes of disability among elderly people and the seventh most common cause of death among all diseases [5]. It is estimated that as many as 6.5 million people have AD [6]. Every five years, the number of AD patients doubles for people above 65 [7,8]. While there is currently no cure for AD, medications like acetylcholinesterase inhibitors can delay cognitive and functional deterioration and may enhance patients’ overall quality of life [9,10]. Therefore, early and accurate diagnosis in the initial stages of AD is crucial to minimize the delay in AD treatment [11]. In addition, an accurate diagnosis is crucial to preventing inappropriate detections with false-positive/false-negative results [12].

The clinical diagnosis of AD usually involves neuroimaging using computed tomography (CT) and magnetic resonance imaging (MRI) [13,14]. However, these neuroimaging methods only deliver a somewhat accurate diagnosis of AD patients. Other techniques, including F-fluorodeoxyglucose positron emission tomography (PET) and single-photon emission computed tomography, are also utilized to diagnose AD and other neurological disorders [15,16]. Amyloid PET is used to diagnose Aβ plaque deposition in the brain cells. However, these techniques are not entirely accurate due to the complexity of the result and their inapplicability in the initial stages of AD [17,18]. Despite neuroimaging, quantitative assays like ELISA and PCR are used to detect amyloid-beta (Aβ) peptides in biological fluids [19,20]. However, these techniques also have limitations for rapid diagnosis and point-of-care testing (POCT) due to the need for sophisticated equipment, trained personnel, long processing times, and complicated operations. Notably, AD usually appears through an abnormal protein produced inside and around brain cells associated with extracellular growth of Aβ plaques. Aβ is produced by the larger amyloid precursor protein (APP) via a proteases-catalyzed hydrolysis process [21]. APP consists of 39–42 amino acids in the fatty membrane surrounding neurons. Most abundant Aβ peptide subtypes, including Aβ_(1-40)_ and Aβ_(1-42)_, are more crucial to the amyloid cascade hypothesis of AD [22]. 

In AD, the levels of Aβ_(1-40)_ and Aβ_(1-42)_ in the brain is higher. The main accumulation of plaques is primarily associated with Aβ_(1-42)_, which can lead to increased free radical damage, reduced immune response, and higher neurotoxicity in the brain. This plaque accumulation, in turn, affects the secretion of pro-inflammatory cytokines and contributes to the formation of neurotoxic oligomers and fibrils, ultimately resulting in severe dementia [17,23]. Additionally, Aβ oligomers (AβO) can interact with the lipid bilayer of neurons, resulting in the disruption of cell membrane homeostasis, more Ca^+^ ions, depolarization of the cell membrane, enhancement of reactive oxygen species, and neuronal excitotoxicity; these reactions ultimately lead to the injury and death of neurons [24]. The normal physiological level of Aβ varies in biological fluids such as blood plasma and cerebrospinal fluid [25,26,27]. In the early stages of AD, an elevation in the total Aβ concentration can be detectable in the preclinical phase before symptoms appear. However, in the advanced stage of AD, Aβ plaques form in the brain cells, and the levels of Aβ (especially Aβ_(1-42)_) usually decrease and return to normal levels [28,29]. Moreover, the Aβ peptide consists of several forms depending on the nucleation process, including amyloid-beta monomer (AβM), AβO, and amyloid-beta fibril (AβF) [30]. Among them, AβO was considered to be strongly correlated with the severity of AD [30]. 

AD commonly develops due to the accumulation of insoluble Aβ peptide and phosphorylated tau proteins, which form tangles of neurofibrils in neurons. The formation of plaques results from the degradation of the amyloid precursor protein (APP) via amyloidogenic and non-amyloidogenic biological processes. In the amyloidogenic process, the production of Aβ peptide occurs through the cleavage of APP by β-secretase. This production leads to the formation of toxic oligomeric species of Aβ peptides, which aggregate and contribute to plaque formation. Conversely, in the non-amyloidogenic process, APP is cleaved by α-secretase, resulting in the prevention of the formation of Aβ and the release of soluble secreted amyloid precursor protein-α (sAPPα). These modifications can potentially disrupt the normal functioning of the brain’s neurotrophic and neuroprotective mechanisms. Overall, the physiological and pathophysiological effects of Aβ peptides play a crucial role in the progression of AD, making them important targets for diagnostic and therapeutic approaches.

Another promising biomarker for the prognosis and diagnosis of AD is the p-tau protein presented in blood with multi-phosphorylation sites. While it is possibly an effective biomarker for the prognosis and diagnosis of AD, a single phosphorylation site may provide false positive results [31,32]. The tau also aggregates within brain cells, creating neurofibrillary tangles. Moreover, it plays a crucial role in binding and stabilizing the microtubules in neuron axons, which is essential for the proper functioning of neurons. However, when tau protein becomes phosphorylated, this process is hindered, resulting in the accumulation of abnormal tau protein which leads to neurofibrillary tangles in brain cells [33]. These tangles develop in specific stages, contributing to the progression of AD. Moreover, abnormal phosphorylation of tau protein is a crucial step in AD development; it leads to the formation of hyperphosphorylated tau protein due to conformational changes in its structure [34]. This abnormal phosphorylation is key to forming both soluble and insoluble tau filaments. The presence of hyperphosphorylated tau protein plays a significant role in the progression of neurodegeneration and AD [35]. An elevated level of total tau (t-tau) and phosphorylated tau (p-tau) in CSF is associated with neurodegeneration and brain damage. Overall, tau protein and its abnormal phosphorylation and aggregation into neurofibrillary tangles are significant factors in the development of AD.

Hence, it is extremely necessary to develop effective methods for the early and accurate detection of AD biomarkers (Aβ and p-tau proteins) [36,37]. One clinical diagnosis against AD is the detection of Aβ _(1-40)_, Aβ _(1-42)_, t-tau, and p-tau_181_ in CSF. However, due to its high cost, invasive method, and lack of availability, AD detection based on CSF biomarkers via the present methods is challenging for broad screening [38,39]. CSF biomarkers have been proven to have relatively high diagnostic accuracy, even for patients with mild cognitive impairment [40]. However, CSF collection causes patient discomfort due to lumbar puncture. Therefore, various studies are reported to develop portable, affordable, and non-invasive quantitative techniques using circulating biofluids such as Aβ_(1-40)_, Aβ_(1-42)_, t-tau, c-tau, p-tau_181_, p-tau_231_, p-tau_381_, and p-tau_441_ [41]. AβO has been considered a highly selective serum biomarker for the early diagnosis of AD [42,43]. 

So far, several new analytical methods have been proposed to detect AD blood biomarkers, including radioimmunoassay [44], ion mobility-mass spectrometry [45], surface plasmon resonance spectrometry [46], surface-enhanced Raman spectroscopy [47], mass spectrometry [48], colorimetric [49], chemiluminescence [50], fluorescence [51], and electrochemical [52,53,54]. All these techniques have advantages and disadvantages; however, many limitations remain to this day. Among these methods, electrochemical techniques have demonstrated rapid response, high sensitivity, easy miniaturization, low cost, and great potential for clinical application [52,53,54,55,56,57,58]. *Electrochemical immunoassay* is a highly sensitive, specific, cost-effective, and rapid technique that can be performed using portable platforms. These advantages make it an attractive alternative to the present clinical diagnostics of AD.

Gold-based and carbon-based nanomaterials can mainly enhance the sensitivity of electrochemical immunosensors [59,60,61,62,63,64,65,66]. These materials have a high surface area-to-volume ratio, which can increase antibody (Ab) immobilization and even enhance the number of binding sites for the analyte, thus improving the sensitivity. 

This review evaluates recent advances (from 2018 to 2023) in electrochemical immunosensors developed to detect the main potential AD biomarkers and provides a comprehensive and thoughtful examination of immunosensor production, including electrode preparation (as the signal transducer), surface modification with nanomaterials for signal amplification, specific biofunctionalization, and a comparison of analytical parameters such as sensitivity, selectivity, reproducibility, detection range, and LOD. The review also aims to explore the immunosensing mechanism and techniques developed to detect AD biomarkers. 

**Figure 1:**
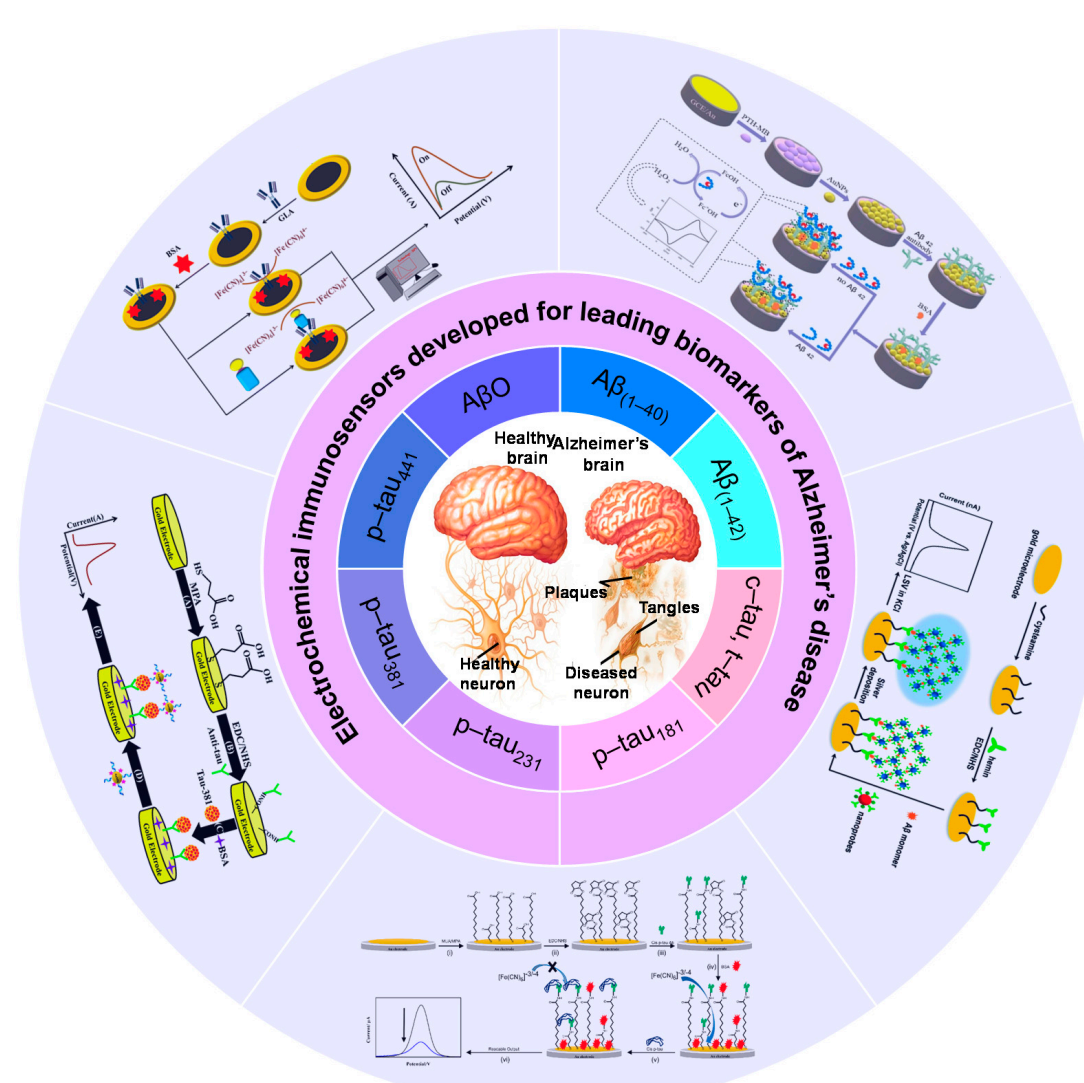
Schematic presentation of electrochemical immunosensors developed for the detection of AD biomarkers.

Figure 1 depicts a schematic illustration of the formation and progression of amyloid plaques in brain cells, which is the leading pathological cause of AD. The illustration also presents specific biomarkers, including Aβ and p-tau protein subtypes, which are utilized for early AD detection. Furthermore, some examples of developed electrochemical immunosensing platforms to detect these biomarkers are presented. 

## 2. Electrochemical Immunosensing of Aβ 

Accurate and early detection of Aβ has numerous significant advantages. First, it enables early AD diagnosis, allowing for prompt intervention and therapy. Delaying disease progression and enhancing patient outcomes are possible with early identification [11,67]. It also allows people to alter their lifestyle and take preventative actions to reduce their risk of getting AD. Second, an accurate and non-invasive way of detecting Aβ is provided by electrochemical biosensors. Electrochemical biosensors have the potential to be portable and less expensive than invasive treatments such as CSF analysis. Even in areas with limited resources, this accessibility enables the extensive screening and monitoring of Aβ levels. Research on AD is also advanced by the ability to detect Aβ using electrochemical biosensors [52,53,54,58]. The development of the disease can be understood by tracking Aβ levels over time, and prospective treatment targets can be found. This information supports creating cutting-edge therapies and exploring new drugs to treat AD and other neurodegenerative diseases. To put it briefly, electrochemical biosensors are crucial for detecting Aβ in clinical and research applications on AD. Electrochemical biosensing platforms allow for early Aβ diagnosis and non-invasive monitoring, which can potentially change AD management and enhance patients’ survival by offering a trustworthy and practical instrument for detection. 

Hao et al., 2022 developed an electrochemical immunosensor based on ZnO nanorod arrays for the selective and rapid detection of Aβ_(1-42)_ [68]. A gold electrode (GE) was produced using photolithography and e-beam evaporation, followed by passivation via polyimide. The resulting electrode was modified with ZnO patterning [69]. Afterward, the surface of the ZnO nanorods was activated via *n,n′*-carbonyldiimidazole molecules and interacted with streptavidin and biotinylated antibodies (Aβ_(1-42)_) via amino groups. Bovine serum albumin (BSA) was used to avoid non-specific adsorption and prevent unwanted immobilization sites. ZnO nanorods provide a high surface-to-volume ratio, rapid electron transfer rate, and good electrical conductivity, allowing the sensitive detection of Aβ_(1-42)_. ZnO nanorod arrays on the GE could improve the electrochemical current response, which increased the detection sensitivity. The differential pulse voltammetry (DPV) response was decreased linearly for Aβ_(1-42)_ concentrations (ranging from 0.5 to 100 pg mL^−1^) and with a low LOD (62.3 fg mL^−1^). The immunosensor performance was also tested with plasma samples of healthy human and AD patients. Considering the behavior of the immunosensor, the DPV peak current related to healthy individuals was higher than in AD patient samples, indicating lower Aβ_(1-42)_ concentrations in AD patient samples than in healthy samples. In addition, the developed immunosensor was also employed to detect p-tau protein in healthy controls and AD patients’ plasma samples; further, it showed remarkable selectivity for both Aβ_(1-42)_ (85.7%) and p-tau (70%). In addition, the immunosensor demonstrated notable stability and reproducibility.

Abbasi et al., 2021 developed an immunosensor for the highly sensitive detection of Aβ_(1-42)_ using conducting polymer-modified graphene electrodes [70]. In order to produce the immunosensing platform, screen-printed graphene electrodes (SPGEs) were electropolymerized in the presence of 1,5-diaminonaphthalene (pDAN) under the optimized condition to create an ultra-thin and uniform layer of polymer. The pDAN-modified graphene electrode surface provided -NH_2_ functional groups, which acted for the attachment of –COOH-functionalized anti-Aβ_(1-42)_ Ab to the graphene surface via the 1-ethyl-3-(3-dimethylaminopropyl)carbodiimide (EDC)/N-hydroxysuccinimide (NHS) crosslinking chemistry. The DPV peak current decreased as the analyte concentrations increased from 1 pg mL^−1^ to 1 ng mL^−1^, and the decrement in DPVs’ peak currents was due to the accumulation of analyte molecules on the electrode surface that blocked the electron transfer between the redox system and the electrode surface. The performance of the immunosensor was also tested with the known concentrations of Aβ_(1-42)_ obtained from plasma samples of AD patients. This demonstrated the potential applicability of the proposed method in clinical assays. 

Wang et al., 2021 proposed an immunosensor for the highly sensitive detection of Aβ_(1–40)_ at low concentrations in human blood and tear fluid [71]. To produce the electrochemical immunosensor, a GE was treated with 11-Mercaptoundecanoic acid (MUA) to create self-assembled monolayers (SAMs), followed by the immobilization of anti-Aβ_(1-40)_ Ab via the EDC/NHS chemistry. Then, various Aβ_(1–40)_ concentrations in the blood and tear fluid sample were then dropped onto the prepared immunosensor and then incubated to ensure specific binding between the Ab and the antigen. The Schirmer strip method was used to collect the human tear fluid samples [72]. In this study, a unique device was designed that consisted of an external centrifuge tube and an internal central filter. The central filter is an essential part of the device used for collecting human tear fluid samples. It plays a critical role in separating the tear fluid sample on the Schirmer strip and prevents the tear fluid from being reabsorbed back into the strips after centrifugation in a tear fluid collection device. The developed immunosensor exhibited the impedance response increase linearly with an increasing concentration of Aβ_(1–40)_, ranging from 1 pg mL^−1^ to 100 pg mL^−1^ due to the binding interaction between the anti-Aβ_(1-40)_ Ab and Aβ_(1-40)_ peptide on the electrode surface, which leads to a change in the electrical properties of the immunosensor’s signal interface. This immunosensor could detect Aβ_(1–40)_ levels as low as 0.1 pg mL^−1^ in human blood serum samples and low as 0.5 pg mL^−1^ in human tear fluid with high sensitivity and good reproducibility (relative standard deviation (RSD) < 6%). 

Wang et al., 2022 developed a sandwich-like immunosensor using a sputtered Au thin film on a 3D nanostructure polycarbonate (PC) substrate to detect Aβ_(1-42)_ monomers/oligomers in blood plasma [73]. The production process of the nanostructured immunosensor involved the preparation of an anodic aluminum oxide (AAO) membrane through an anodizing process using the phosphoric acid (etchant), which formed a uniform hemisphere structure with a diameter of 400 nm and a height of 75 nm. The 3D nanostructure was then transferred from the AAO membrane to the nickel mold. This nickel mold was used to replicate the 3D nanostructure on a PC template through hot embossing, followed by depositing Au film (thickness: 30 nm) on the 3D nanostructure PC substrate. Afterward, a uniform layer of AuNPs (diameter: 10–15 nm) was deposited on the electrode surface via an electrochemical method, which increased the surface area of the immunosensor and provided a more accessible area for assembling SAM molecules, thereby increasing the detection sensitivity. Additionally, the AuNPs acted as a signal amplifier and improved the LOD of the immunosensor. The Au surface was modified with a SAM layer of MUA to provide desired binding sites for immobilizing a monoclonal Ab (12F4) via the EDC/NHS chemistry. Then, antigen (Aβ _(1-42)_ monomers and oligomers) concentrations were added to the Ab-modified surface and interacted with Ab molecules. Afterward, another Ab (probe Ab) was added, and the sensing platform was completed as a sandwich-like structure. The electrochemical impedance spectroscopy (EIS) assay showed that the impedance increased linearly with increasing concentrations of Aβ_(1-42)_ (ranging from 10 pg mL^−1^ to 100 ng mL^−1^), with a LOD of 113 fg mL^−1^. The presented sandwich-like immunosensor exhibited good selectivity, reproducibility, and stability. It also showed a short preparation time of 1.5 h and a rapid detection time of 2 min, significantly faster than ELISA and western blot analyses. 

Tehrani et al., 2021, reported another immunosensor for detecting Aβ_(1-42)_ using a diazotized GCE [74]. The surface of the GCE was electrochemically functionalized with an amino group (-NH_2_) using cyclic voltammetry (CV) in a mixture solution of diazonium salt in an electrolyte of BU_4_NBF_4_ and acetonitrile. Then, the electrochemically activated ferrocene carboxylic acid (FCA) was deposited on the electrode surface, and through the EDC/NHS chemistry, it could be linked to the -NH_2_-modified GCE surface. Afterward, the -COOH functionalized anti-Aβ_(1-42)_ Ab was immobilized on the electrode surface, and then the prepared immunosensor was incubated with the various concentrations of Aβ_(1-42)_ peptide. DPVs’ peak currents decreased linearly with increasing concentrations of Aβ_(1-42)_, ranging from 10 ng mL^−1^ to 200 ng mL^−1^, with the high sensitivity 5.07 µA/ng mL^−1^. The aminophenyl bonding sites on the diazotized GCE surface played a crucial role in binding the Ab molecules, which is influenced by the diazonium functionalization efficiency. In addition, modifying the electrode surface with amino groups caused the easier trapping of the Ab molecules, thereby improving the immunosensor sensitivity.

Kasturi et al., 2021 developed a miniaturized immunosensor integrated with microfluidic channels for the electrochemical detection of Aβ_(1-42)_ [75]. The miniaturized device consisted of integrated microelectrodes (Au/Ag/Au) configuration with the microfluidic channel and valve-control system to precisely control the liquid flow for the controlled immobilization of biomolecules onto the Au microelectrode. The microelectrodes (Au/Ag/Au) were produced via photolithography and sputtering. The developed immunosensor detected the formation of Ab-antigen complexes on the Au microelectrode integrated inside microfluidic chambers. The microfluidic immunosensor exhibited a linear relationship between the detected signal and Aβ_(1-42)_ concentrations in a linear range from 2.2 pM to 22 μM., with a LOD of 1.62 pM. The DPV measurements revealed that the peak current (I_pc_) values decreased significantly as the concentration of Aβ_(1-42)_ increased. This phenomenon occurred due to the formation of an Ab-antigen complex on the electrode surface, which hindered electron transport on the Au surface. The miniaturized immunosensor integrated with microfluidic channels introduced several advantages, including high sensitivity and selectivity, precise control of biological sampling with fewer production costs and low sample volumes requiring, and even elimination of the laborious pipetting requirement, which maybe reduce waste and sampling time. 

Ding et al., 2021, reported an Aβ_(1-42)_ immunosensor using a heme-Aβ_(1-42)_ conjugated structure as a signal amplifier to detect the analyte in serum and saliva [76]. A GCE (diameter: 3 mm) was activated using CV in 0.5 M H_2_SO_4_ under potential from −0.5 to 1.6 V. Then, the electrode surface was modified with AuNPs through the electrodeposition of a HAuCl_4_ solution via the CV technique. Afterward, AuNPs (diameter: ∼45 nm) modified GCE electrode was modified with thionine (Thi) and methylene blue (Mb). Subsequently, one more layer of AuNPs was deposited to produce GCE/Au/pTh-Mb/AuNPs electrode. The modified electrode was then functionalized with self-assembled monolayers of alpha-lipoic acid (ALA) and the -COOH group of the ALA layer, which was activated via the EDC/NHS chemistry for covalent binding to anti-Aβ_(1-42)_ Ab on the electrode surface. The conjugated form of heme and Aβ_(1-42)_ was prepared by mixing the appropriate amounts of each (1:1) in 0.1 NaOH to enhance the electrocatalytic process of H_2_O_2_ in the presence of ferrocene methanol (FcOH), resulting in the oxidation of FcOH, to produce Fc^+^OH. The electrochemical response was measured using CV, and the reduction peak current (I_pc_) was decreased with an increase in the concentration of Aβ_(1-42)_ due to the reduction of FcOH/Fc^+^OH catalyzed by anti-Aβ_(1-42)_ modified heme-Aβ_(1-42)_. The presented immunosensor exhibited good linearity over the range of 0.056–13.7 nM (in fetal bovine serum), 0.056–41.2 nM (in artificial saliva), with a LOD of 25.2 (fetal bovine serum), and 23.8 (artificial saliva). In addition, the immunosensor showed a good selectivity towards Aβ_(1-42)_ peptide against human serum albumin and lysozyme; the stability was found to be up to 28 days and 87.4% of the initial performance was retained.

**Figure 2:**
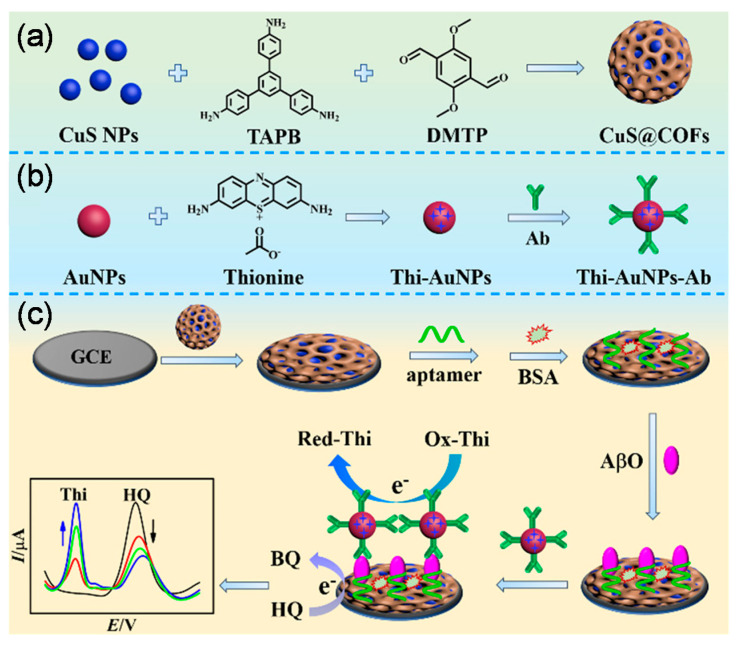
Schematic representation of the synthesis of CuSNPs@COFs nanocomposite (**a**); producing AuNPs conjugated with Thi and Ab (**b**); and production of the ratiometric sandwich-like electrochemical immunosensor on a GCE (**c**). This figure is reprinted from [61] with permission from the American Chemical Society.

Chen et al., 2022 proposed a sandwich-like immunosensor to detect AβO using a platform equipped with Thi and gold nanoparticles (Thi-AuNPs) and copper sulfide nanoparticle-modified covalent organic framework (CuSNPs@COFs) hybrid nanocomposite [77]. To synthesize the CuSNPs@COFs nanocomposite, copper (II) chloride (CuCl_2_) and sodium citrate were dispersed in the DI water under continuous stirring, followed by adding sodium sulfide, and then reacting overnight at room temperature to attain CuSNPs (diameter: 13 nm). To encapsulate the CuSNPs with COFs, CuSNPs and 1,3,5-tris(4-aminophenyl) benzene were mixed in *n*-butanol, acetic acid, 2,5-dimethoxyterephthalaldehyde, and 4-ethylene oxide under continuous stirring. The resulting CuSNPs@COFs nanocomposite was washed with butanol, acetone, and DI water, which was subsequently dried in a vacuum overnight (Figure 2a). In order to synthesize the Thi-AuNPs-Ab conjugated form, AuNPs were synthesized using a HAuCl_4_ solution mixed with sodium citrate, and the achieved mixture was boiled for 10 min under vigorous stirring. AuNPs were produced after filtering and drying under optimum conditions. Afterward, a Thi solution was added to the AuNPs solution in a ratio of (1.1 v%) and kept in the optimized reaction for 24 h, followed by washing with PBS. Then the anti-Aβ Ab was added to the Thi-AuNPs solution (Figure 2b). In order to design the sensor interface, a GCE was modified with CuSNPs@COFs, followed by treatment with a ssDNA aptamer, while non-specific binding sites were eliminated by adding a BSA solution under optimized conditions. Afterward, the electrode surface was incubated with different concentrations of AβO and then treated with the Thi-AuNPs-Ab conjugated form, forming a sandwich-like (aptamer/analyte/Ab) complex (Figure 2c). The DPV responses were recorded for linear concentrations of AβO from 1 pM to 1 μM, resulting in decreased hydroquinone (HQ) responses while the Thi signals inversely increased. The presence of analyte molecules was the reason for the connection of Th molecules to the electrode surface. The calibration plot showed that the double peak (IThi/IHQ) signal ratio steadily increased with the increasing AβO concentrations, and the estimated LOD was about 0.4 pM. This immunosensor showed satisfactory sensitivity toward AβO molecules in CSF samples. Further, the results demonstrated the desired selectivity toward AβO, and no significant response was found against interfering substances (AβM, AβF, human serum albumin (HSA), BSA, tryptophan, and glycine). Moreover, the developed immunosensor demonstrated excellent reproducibility with RSD 2.5% for five times assay and good stability for up to two weeks. 

Supraja et al., 2021, reported a label-free and highly sensitive detection of Aβ_(1-42)_ peptide in human plasma using an immunosensing platform equipped with SnO_2_ nanofibers (SNFs) [78]. In order to produce the sensor interface, SNFs were synthesized via electrospinning using a uniform mixture of polyacrylonitrile and dimethylformamide (DMF), followed by adding tin (IV) acetate under constant stirring. The attained mixture was an electrospun with a flow rate and an electric field between a metallic needle’s tip and a collector plate. The final product was subjected to a calcination process, producing solid SNFs powder. Afterward, the SNF suspension dissolved in DMF was drop-casted onto a GCE (diameter: 3 mm) surface and dried under optimal conditions. Then, the surface of GCE/SNFs was functionalized with 3-Mercapto-propanoic acid (MPA) via electrostatic interaction between sulphydryl groups of MPA and Sn^+4^ ions of SNFs and subsequently, MPA molecules on the electrode surface was activated via the EDC/NHS chemistry. The activated GCE/SNF was treated with anti-Aβ_(1-42)_ Ab via the covalent binding to form the stable amide bond (CO-NH). The established immunosensor could detect the Aβ_(1-42)_ peptide concentrations in a range from 1 fg mL^−1^ to 1 ng mL^−1^ in human plasma, and good linearity was found between charge transfer resistance (R_ct_) vs. Aβ_(1-42)_ peptide concentrations. The reported LOD was about 0.638 fg mL^−1^. The values of R_ct_ increased with increasing analyte concentrations, which was attributed to changes in the electrical properties of the electrode surface caused by the binding of Aβ_(1-42)_ peptides to the immobilized capture Ab molecules, which repelled the redox marker molecules from the electrode surface. The authors of this research claimed that this immunosensor obtained long-term stability up to the 126th day, the desired reproducibility, and excellent selectivity against various interfering substances (Aβ_(1-40)_, BSA, HSA, Troponins, etc.).

**Figure 3:**
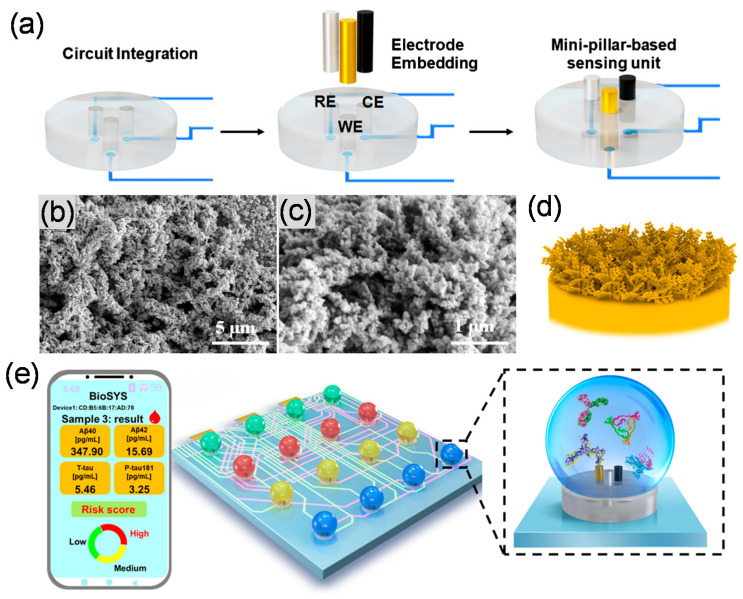
Schematic illustration of a mini-pillar-based portable electrochemical sensing platform for the detection of multiple AD biomarkers (**a**); SEM micrographs of the Au nanostructure (**b**,**c**); schematic architecture of the electrode surface modified with the Au nanostructure (**d**); and the portable platform for detection of multiple AD biomarkers (Aβ_(1-40)_, Aβ_(1-42)_, and p-tau_181_) (**e**). Reprinted from [79] with permission from Springer Nature.

Liu et al., 2022 developed an electrochemical immunosensor array integrated with a portable micro-workstation platform to detect multiple AD biomarkers, including Aβ_(1-40)_, Aβ_(1-42)_, and p-tau_181_ [79]. To produce the sensing platform, a polytetrafluoroethylene template was coated with a mixture of polydimethylsiloxane (PDMS) and a curing agent (16:1), followed by the embedding of all three electrodes (working electrode (WE): Au,; counter electrode (CE): a Pt wire; and reference electrode (RE): Ag/AgCl) into the PDMS layer. All three electrodes were embedded in the mini-pillar, and the bottom of the electrode wires was connected to an integrated circuit board. The complete assembly process for this platform is presented in Figure 3a. Then, an array of Au nanostructure was electrodeposited using HAuCl_4_ solution (−1.4 V) on the WE, and scanning electron microscopy (SEM) micrographs were provided to characterize the morphology of the synthesized Au nanostructure (Figure 3b–d), which confirms a nano-dendritic shape on the electrode surface. In the next step, the specific Ab for each protein Aβ_(1-40)_, Aβ(_1-42)_, and p-tau_181_ was immobilized onto the surface of the WE/Au nanostructure, and the BSA was used as the blocking agent to eliminate the non-specific Abs binding sites. The analytes concentrations (Aβ_(1-40)_, Aβ_(1-42)_, and p-tau_181_) were incubated on the electrode surface, followed by horseradish peroxidase (HRP)-labeled secondary Abs immobilized onto the electrode surface. The HRP catalyzes the breakdown of H_2_O_2_, which causes an enhancement of the current values. The presented immunosensor was connected to a portable electrochemical micro-workstation compatible with smartphones via Bluetooth. This platform was capable of detecting multiple AD biomarkers (Figure 3e). The results showed a good analytical performance, such as linearity, for each protein (Aβ_(1-40)_, Aβ_(1-42)_, and p-tau_181_) concentration range (0.1 to 1000 pg mL^−1^), and the LOD found for Aβ_(1-40)_ (0.125 pg mL^−1^), Aβ_(1-42)_ (0.089 pg mL^−1^), and p-tau_181_ (0.176 pg mL^−1^), along with the desired selectivity and stability. 

Table 1 presents the primary features of recently developed electrochemical immunosensors for detecting Aβ peptides.

## 3. Electrochemical Sensing of Tau Protein

Neuronal neurofibrillary tangles created by tau protein impede cellular function and impair cognition. It is crucial to identify tau protein using electrochemical biosensors. This enables early and precise identification of AD, assisting with prompt therapy and intervention [94,95]. As a powerful biomarker for disease development and therapy response, tau protein detection also enables therapeutic efficacy monitoring. Additionally, measuring tau protein concentrations with electrochemical biosensors advances our knowledge of the mechanisms underlying AD [96,97,98]. Researchers learn more about the course of the disease, the dissemination of tau pathology, and prospective treatment targets by examining tau protein dynamics. Electrochemical biosensors are fundamental for detecting tau protein in clinical and research paths. It makes early diagnosis easier, makes it possible to track the development of the disease, and improves the understanding of the underlying mechanisms.

Le et al., 2021 developed an immunosensor for the detection of phosphorylated-tau threonine 231 (p-tau_231_) using an interdigitated wave-shaped electrode (IWE) for analyzing the analyte in human serum [99]. The IWE (14 × 3.5 mm) (Ti/Au) was first modified with a SAM of 6-mercaptohexanoic acid. Subsequently, using the EDC/NHS chemistry provided active binding sites for anti-p-tau_231_ Ab. During the analyte detection, the R_ct_ enhanced with increasing concentrations of p-tau_231_ in a linear range from 10^−4^ to 10^1^ ng mL^−1^ in human serum, which could reach the LOD of 140 pg mL^−1^. The concentration of p-tau_231_ in the CSF of AD patients is higher (700 pg mL^−1^) than the sensor’s LOD, showing the immunosensor’s potential for detecting p-tau_231_ in early-stage AD. This immunosensor exhibited a high binding affinity between anti-p-tau_231_ and p-tau_231_ with a low dissociation constant (K_d_: 7.6 pM) in human serum, demonstrating the highly selective detection of p-tau_231_. The stability was maintained for up to two weeks.

Chen et al., 2022, reported a hydrogel-based immunosensing platform based on loach mucus-inspired guanosine-based hydrogel (G4 hydrogel), providing a high antifouling performance for the selective detection of tau protein [100]. A GCE was modified via electrodeposition of a cationic polymer, e.g., poly dimethyl diallyl ammonium chloride (PDDA), under a constant potential to construct the antifouling interface on the electrode surface. The surface of the GCE was then modified via drop-casting of a G4 hydrogel solution onto the PDDA-modified GCE, which, due to the electrostatic interaction, could produce a compact film on the electrode interface. G4 hydrogels were prepared by heating 3-carboxyphenylboronic acid and guanosine (1:1 M ratio) in a KOH solution. Then, the mixture was cooled to ambient temperature. In the presence of alkali metal ions, guanosine (G) can be self-associated to form macrocyclic tetramers known as G-quarters. Several G-quartets stacked through hydrogen bonds to form G-quadruplexes, which then formed the guanosine hydrogel. The final electrode surface was employed for specific immobilization of the tau Ab. The prepared hydrogel-based immunosensor in the presence of various analyte concentrations exhibited a high selectivity and sensitivity in a linear range from 0.01 ng mL^−1^ to 100 ng mL^−1^, and the estimated LOD was about 1.31 pg mL^−1^. The critical tau protein concentration (0.2 ng mL^−1^) differentiating AD cases is lower than the obtained LOD. Here, the biocompatible and highly hydrophilic G4 hydrogel could increase specificity and sensitivity due to creating a thick coating to avoid antifouling on the GCE surface, which created a hydration barrier, providing exceptional resistance to non-specific adsorption in high ionic strength biofluids (i.e., whole blood, plasma).

**Figure 4:**
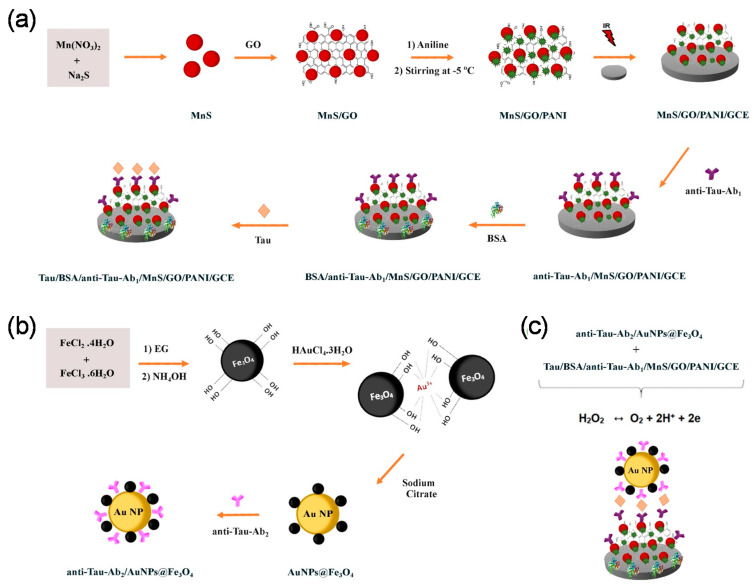
Schematic representation of an electrochemical immunosensor developed to detect the tau protein; chemical synthesis of the MnS-GO-PANI nanocomposite and production of the immunosensor platform (**a**); synthesis of AuNPs and Fe_3_O_4_NPs in a conjugated form with anti-tau-Ab_2_ (**b**); and the immunosensing platform was formed by the interaction of GCE/PANI/GO/MnS/anti-tau-Ab_1_/analyte with anti-tau-Ab_2_/Au@Fe_3_O_4_NPs through specific Ab-antigen interactions (**c**). Reprinted from [101] with permission from Wiley.

In another study, Yola et al., 2022, developed an immunosensor for the tau protein using manganese sulfide nanoparticles/graphene oxide/polyaniline (MnS/GO/polyaniline (PANI)) and magnetite-decorated Au nanoparticles (Fe_3_O_4_/AuNPs) as a signal amplifier [101]. To synthesize MnS/GO/PANI nanocomposite, a manganese nitrate (Mn(NO_3_)_2_) solution dissolved in the DI water was slowly mixed with the Na_2_S solution at 25 °C for 60 min, followed by adding GO (synthesized via the modified Hummers’ method). The final dark brown precipitate (MnS/GO) resulted in the growth of MnS NPs (diameter: 20–25 nm) on the GO sheet. The synthesized (MnS/GO) was then slowly mixed with aniline and ammonium persulphate ((NH_4_)_2_S_2_O_8_) and kept at −5 °C for 4 h to produce the homogeneous suspension. The final product (MnS/GO/PANI) was washed with methanol and DI water (Figure 4a). The MnS-GO nanocomposite contained many active sites suitable to enhance the nucleation reaction for the polymerization of aniline molecules. These aniline molecules were electrostatically reacted with functional groups (-COOH and -OH) of GO, and these aniline molecules used to be polymerized on the MnS-GO nanocomposite surface. Therefore, the MnS-GO-PANI nanocomposite showed high stability through an effective electrostatic interaction of MnS NPs and GO and a covalent interaction related to the MnS-GO and PANI. For the synthesis of the AuNPs@Fe_3_O_4_/anti-tau-Ab_2_ conjugated form, the mixtures of FeCl_2_·4H_2_O and FeCl_3_·6H_2_O were dissolved in a solution of ethylene glycol and DI water at 70 °C under continuous stirring, followed by slowly adding the NH_4_OH. The Fe_3_O_4_NPs (diameter: 10–15 nm) were obtained after washing with DI water. Afterward, the HAuCl_4_·3H_2_O and ethylene glycol mixture was added to the Fe_3_O_4_NPs solution under stirring at 90 °C for 5 min. Then, the achieved mixture was added to the sodium citrate solution under further stirring for 30 min to reach the AuNPs-Fe_3_O_4_ nanocomposite. The anti-tau-Ab_2_ was incubated with the AuNPs-Fe_3_O_4_ nanocomposite to have AuNPs/Fe_3_O_4_-anti-tau-Ab_2_ conjugated form (Figure 4b). To produce the immunosensor, MnS-GO-PANI nanocomposite solution was drop-casted onto a GCE surface and dried under an infrared heat lamp for 30 min, followed by immobilization of anti-tau-Ab_1_, strongly attached via stable electrostatic/ionic interactions. Then, various concentrations of the analyte were added to the surface of the final electrode (GCE/PANI/GO/MnS/anti-tau-Ab_1_). As the last step, the AuNPs/Fe_3_O_4_/anti-tau-Ab_2_ conjugated form was immobilized as the signal amplifier (Figure 4c). The electrochemical assay demonstrated that DPV signals increased with increasing the tau protein concentrations in a linear range from 1 × 10^−13^ to 1 × 10^−6^ M, with a LOD of 1 × 10^−14^ M. The presented sandwich-like immunosensing platform exhibited high sensitivity and a linear relationship with tau protein due to its special assembly and the presence of AuNPs (diameter: ~68–75 nm) and Fe_3_O_4_ NPs (diameter: ~10–15 nm), which provided a high surface area, rapid electron transfer, and a strong synergistic effect. This resulted in the enhancement of the electrochemical activity. 

Schneider et al., 2022, reported an immunosensor to detect phosphorylated tau_181_ (p-tau_181_) protein in serum using a SPCE modified with a nanocomposite containing multi-walled carbon nanotubes and platinum nanoparticles (MWCNTs/PtNPs) [102]. MWCNTs (diameter: 10–15 nm) were dissolved in an aqueous NaCl solution that contained 1 wt% polyallylamine hydrochloride (PAH) and under constant enough magnetically stirring; the mixture was then subjected to sonication with an ultrasonic probe. The resulting mixture was centrifuged to remove the excess PAH, and the DI water was used to disperse it again via sonication. To synthesize the PtNPs, the sodium borohydride (NaBH_4_) was slowly introduced into a solution of chloroplatinic acid hydrate and sodium citrate tribasic dihydrate in water (molar ratios: Na_3_C_6_H_5_O_7_/H_2_PtCl_6_/NaBH_4_ 2:1:0.3) at room temperature. Finally, the brown-colored product of dendritic PtNPs (diameter: 2 to 5 nm) was obtained after vigorous stirring at room temperature. Afterward, MWCNTs-PAH solution and PtNPs were mixed via continuous stirring and some centrifugations, and the resulting nanocomposite was then dispersed in the DI water. To produce the immunosensor, the nanocomposite (MWCNTs-PtNPs) was drop-casted on a SPCE (diameter: 4 mm) and dried at 60 °C for 30 min. Then, the anti-p-tau_181_ Ab was immobilized onto the nanocomposite-modified SPCE. The SWV response was used to measure the electrochemical behavior, and the results showed that with increasing concentrations of p-tau_181_ in a linear range from 8.6 to 1100 pg mL^−1^, a decrease in the peak currents was found (signal-off). The reported LOD for this immunosensor was about 0.24 pg mL^−1^. The decrease in peak currents was observed due to the binding of p-tau_181_ to the immunosensor surface, which blocked the electron transfer pathways necessary for current flow on the sensing surface. This immunosensor exhibited good reproducibility and selectivity against several interference substances, such as uric acid, IgG, hemoglobin, and BSA.

Sonuç Karaboğa et al., 2022, proposed a disposable and portable immunosensor for detecting p-tau_441_ protein [103]. In order to produce the immunosensing platform, a SPCE was treated with 3-Aminopropyl)triethoxysilane in ethanol, followed by rinsing with ethanol, and then heated at the optimal temperature. The SAM-modified SPCE was cross-linked via glutaraldehyde. Under optimal conditions, an anti-p-tau_441_ Ab was immobilized on the glutaraldehyde-crosslinked SPCE. The EIS showed the formation of the insulating protein layer on the electrode surface, leading to an increase in R_ct_, which allowed the partial diffusion of the redox probe [Fe(CN)_6_]^3−/4−^. The proposed immunosensor demonstrated satisfactory linearity for p-tau_441_ protein in a linear range from 0.0064 to 0.8 ng mL^−1^, and the estimated LOD was about 0.0053 ng mL^−1^. In addition, the immunosensor identified the p-tau_441_ protein in the patient’s CSF samples.

**Figure 5:**
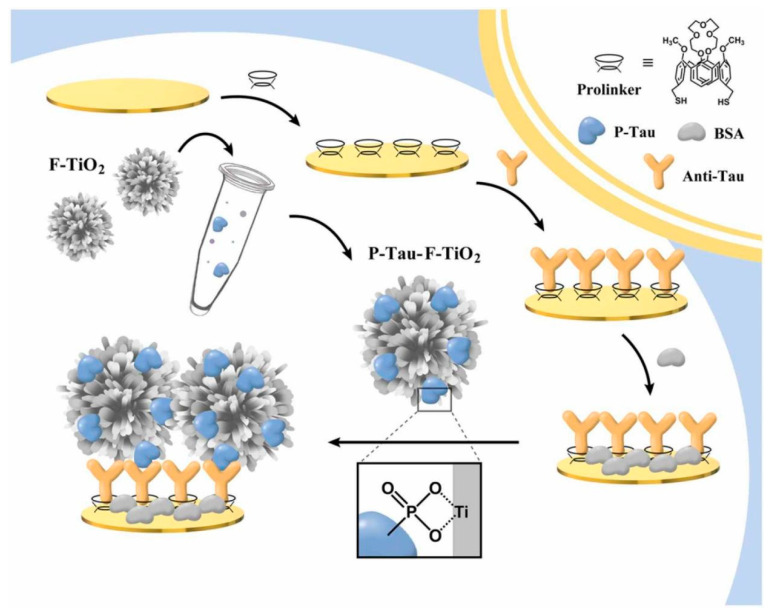
Schematic illustration of the flower-shaped TiO_2_-based electrochemical immunosensor for detecting p-tau protein. Reprinted from [31] with permission from Elsevier.

In another study, Zhang et al., 2022, developed an immunoassay platform using flower-shaped titanium oxide (TiO_2_) as a signal amplifier for the selective detection of p-tau [31]. First, a GE was cleaned using a piranha solution, and the electrode surface was activated further via CV in 0.5 M H_2_SO_4_ solution (by applying 0–1.6 V) to remove any probable unwanted molecules. Then, a ProLinker (Calixarene derivative) in chloroform–methanol solution (1:3, *v*/*v*) was pipetted on the electrode surface and left to react overnight at room temperature. Afterward, the anti-tau Ab solution was added to the GE/ProLinker to form the sensing layer, and the unreacted sites were blocked by BSA. Afterward, the flower-shaped TiO_2_ suspension was mixed with different concentrations of p-tau protein (*v*/*v*, 1:1), drop-casted on the electrode surface, and reacted with the sensing surface for 1 h. To synthesize the flower-shaped TiO_2_, tetrabutyl titanate was mixed with the DI water and HCl under continuous stirring for 1 h. The achieved mixture was moved to a Teflon-lined autoclave and heated at 160 °C under controlled conditions. The final product was obtained after washing and centrifugation. Figure 5 shows the processes for fabricating the developed immunoassay platform for detecting p-tau protein. The detection performance was evaluated using the EIS technique. The value of R_ct_ was increased with increasing the concentration of the p-tau protein. The immunoassay showed good sensitivity, with a LOD of 1.774 pg mL^−1^. Due to the binding between the phosphoric acid group of the p-tau protein and TiO_2_ (p-tau-F-TiO_2_ complex), this complex could be captured onto the anti-tau Ab-treated surface, and the desired sensing performance of the immunosensor was attained. The reported linear detection range for this immunosensor is from 1 to 200 ng mL^−1^.

Khetani et al., 2021, developed a highly sensitive immunoassay platform to detect multiple proteins, such as c-tau protein and neuron filament (NFL), in the blood of traumatic brain injury patients [104]. To produce the sensor interface, all three electrodes (WE: Ti/Au), (CE: Ti/Au), and (RE: Pt) were patterned by sputtering onto a glass substrate using polymethyl methacrylate shadow masks, followed by annealing under optimized temperature. Afterward, the WE electrode was modified with a SAM layer of MUA in an ethanol solution under N_2_. The MUA-modified WE electrode was activated with the EDC/NHS chemistry to bind the antibodies for specific proteins via amino functional groups. The DPV response was measured for the c-tau and NFL concentrations ranging from 1 μg mL^−1^ to 1 pg mL^−1^. The immunoassay showed a decrease in DPVs peak currents with increasing concentrations of both analytes (c-tau and NFL), and this behavior may be due to the formation of insulating protein layers causing decreased diffusion of the redox probes on the electrode surface. The measured LOD with a potentiostat was to be 0.34 pg mL^−1^ for c-tau and 0.2 pg mL^−1^ for NFL, and it was 0.14 pg mL^−1^ for c-tau and 0.16 pg mL^−1^ for NFL when measured by another potentiostat (μDrop potentiostat). The authors of this study claimed that immunoassay by the μDrop is suitable for the sensitive detection of c-tau and NFL biomarkers that could obtain a more satisfactory LOD than the commercial potentiostat.

Hun et al., 2021, proposed a photoelectrochemical (PEC) biosensor for the detection of p-tau_381_ protein using transition-metal dichalcogenides (selenide molybdenum, MoSe_2_) modified AuNPs (AuNPs/MoSe_2_) [105]. MoSe_2_ nanosheets (NSs) were produced via an ultrasonic exfoliation process from a bulk part of MoSe_2_ dissolved in DMF; then, the obtained mixture was centrifuged and rinsed with ethanol several times to remove any impurities and unwanted solvent. To decorate the AuNPs on MoSe_2_ nanosheets, HAuCl_4_ solution and MoSe_2_NSs (1:1 v%) were mixed and boiled with continued vigorous stirring; then, the sodium citrate was added slowly under continuous stirring. AuNPs-MoSe_2_NSs nanocomposite was obtained after washing several times with DMF. The AuNPs-MoSe_2_NSs nanocomposite solution was drop-casted on a carbon electrode and dried at optimized conditions. Afterward, a thiol-functionalized DNA aptamer was immobilized onto the nano-modified carbon electrode, bound via the Au-S bond. Then, the prepared biointerface was incubated with different concentrations of p-tau381 protein under optimized conditions to form aptamer-analyte interaction. Then, the tau Ab was immobilized and this biomolecule could capture the analyte from the other side. Subsequently, the biosensor was treated with protein G/AP (recombinant protein G labeled with alkaline phosphatase) under optimized conditions. As the last step, the biosensor was immersed into a mixture solution of magnesium nitrate (Mg(NO_3_)_2_) and ascorbate alkaline phosphate (AAP as an enzyme catalyst) (v% 1:1) in 0.1 M Tris-HCl and incubated at 37 °C to produce an efficient PEC response. The high PEC response of AuNPs-MoSe_2_NSs/CE was achieved after modifying with enzyme (AAP), G/AP protein, and anti-p-tau381 Ab/protein/aptamer complex. The photocurrent was increased with increasing concentrations of p-tau381 in a linear range from 0.5 fM to 1 nM, and the obtained LOD was about 0.3 fM. This biosensor showed excellent selectivity against interfering substances such as tau_410_, tau_441_, alpha-1 antitrypsin, and HAS. Notably, the stability found was up to 28 days, whereas the photocurrent response slightly decreased by 7.2% compared to the first day.

Karabiga et al., 2020, developed an immunosensor for the p-tau_441_ protein detection using AuNPs-modified-rGO on an indium tin oxide (ITO) electrode-coated polyethyetheraphthalate disposable electrodes [106]. To form active-hydroxyl (-OH) groups, the electrode was immersed in a mixture solution of NH_4_OH, H_2_O_2_, and DI water (1:1:5 *v*/*v*) under optimized conditions, followed by rinsing the electrode with the deionized water. Afterward, the electrode surface was coated with the rGO suspension dispersed in the DMF. In another step, AuNPs were electrodeposited on the rGO-modified ITO electrode, and then the final electrode was treated with the MUA solution in ethanol overnight. The carboxylic acid groups related to the MUA-modified electrode were activated with the EDC/NHS chemistry to create binding with amino groups of the anti-p-tau_441_ Ab. The prepared immunosensor was incubated with different concentrations of p-tau_441_ protein, and the interaction between the immunosensor and analyte was evaluated by CV and EIS techniques. The Rct values were increasing, while CVs anodic and cathodic peak currents decreased with enhancing concentrations of p-tau_441_ protein in a linear range from 1 to 500 pg mL^−1^ due to the efficient binding of the Ab-antigen immunocomplex, causing a kinetic barrier that interrupts the interfacial electron transfer at the electrode surface. The presented immunoassay showed the LOD to be 0.091 pg mL^−1^. 

Li et al., 2021, produced an immunoassay system for detecting p-tau_441_ protein using MWCNT_S_-rGO-chitosan (CS) nanocomposite and AuNPs as signal amplifiers [107]. The MWCNTs-rGO-CS nanocomposite was synthesized from an appropriate amount of MWCNTs, rGO, and CS added to an acetic acid solution. Then, the glutaric dialdehyde (GLA) was mixed with the MWCNTs-rGO-CS nanocomposite, and the resultant mixture was drop-casted on a GE surface; a thin layer of the MWCNTs-rGO-CS-GLA nanocomposite was formed onto the electrode surface via the electrostatic interaction. Afterward, the anti-p-tau_441_ Ab was immobilized onto the electrode surface. The presence of nanocomposite on the GE surface enhanced the electrical property that could improve the electron transfer between redox marker molecules and the electrode surface. The immobilization process of the Ab on the electrode surface was due to aldehyde groups at both ends of GLA covalently binding with amino groups of the CS and Ab molecules. In order to produce the immunosensor, the GE surface was treated with KOH, H_2_SO_4_, and HNO_3_, respectively, and further electrochemically activated (0.5 M H_2_SO_4_) via CV to eliminate the probably oxidized film and any other organic residue molecules from the electrode surface. The HAuCl_4_ and sodium citrate solution were mixed with ultrapure water under a constant magnetic string, and then, the NaBH_4_ solution was added slowly under continuous stirring for 2 h. The orange color of the AuNPs solution (diameter: 4 nm) was obtained, and these NPs were mixed with the cysteamine under ultrasonic heating at 50 °C. Finally, the AuNPs-cysteamine conjugated form was mixed with different concentrations of p-tau_441_ protein at 1:2 v% and subsequently incubated under the optimized condition to make the AuNPs-p-tau_441_ complex. Thiol groups (-SH) at one end of the cysteamine could create the Au-S bond with AuNPs, and the other end contained amino groups, which could be attached to the p-tau441 protein. The AuNPs-p-tau441 complex could be detected selectively after immobilization of specific Ab. The DPV technique was used for the immunoassay; the peak currents decreased linearly with increasing p-tau441 protein concentrations ranging from 0.5 to 80 fM, and the achieved LOD was about 0.46 fM. The results demonstrated that the formation of a complex between the AuNPs-p-tau441 protein and the GE/rGO-CS/MWCNTs/Ab surface hindered the main electron transfer, which decreased the DPV signal. The immunosensor also showed satisfactory selectivity against several interfering agents, including glucose, ascorbic acid, L-cysteine, HSA, and α-synuclein.

Huang et al., 2022, developed a highly wettable immunosensor to detect multiple Aβ_(1-40)_, Aβ_(1-42)_, p-tau_181_, and t-tau [91]. Here, vertical graphene was grown onto a ceramic surface via chemical vapor deposition, and then, AuNPs were decorated on the graphene surface using HAuCl_4_ by applying a constant potential (−1.8 V). The AuNPs modified graphene substrate was first treated with n-decanethiol solution and then treated with O_2_ plasma to obtain a super wetting electrochemical substrate. Afterward, selective antibodies against target proteins (Aβ_(1-40)_, Aβ_(1-42)_, p-tau_181_, and t-tau) were pipetted onto the super hydrophilic AuNPs/VG surface and then incubated with analytes at 37 °C. The peak currents of DPVs were decreased with increasing concentrations of analytes in a linear range from 0.1 pg mL^−1^ to 1 ng mL^−1^, and the estimated LOD for Aβ_(1-40)_, Aβ_(1-42)_, p-tau_181_, and t-tau was 0.064 pg mL^−1^, 0.012 pg mL^−1^, 0.041 pg mL^−1^, and 0.039 pg mL^−1^, respectively. The decrease in DPVs peak currents was attributed to the successful attachment of analyte molecules on the signal transducer’s surface, reducing the accessible spatial area for redox marker molecules.

Table 2 presents the primary features of recently developed electrochemical immunosensors for detecting tau proteins. 

## 4. The Fundamental Elements Involved in the Design and Development of Electrochemical Immunosensors for the Detection of AD Biomarkers 

**Figure 6:**
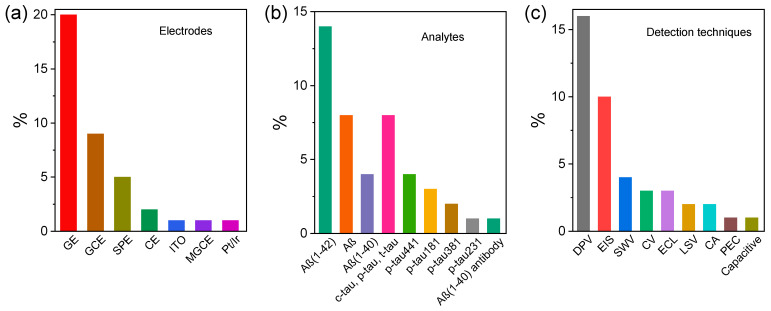
Statistics based on Table 1 and Table 2 about key characteristics of recent electrochemical immunosensors developed for detecting AD biomarkers (Aβ and tau proteins) using different electrochemical techniques; various types of electrode materials used as the signal transducer (**a**); frequency of different AD biomarkers (**b**); and various employed electrochemical detection techniques (**c**).

The literature summary in Table 1 and Table 2 provides an overview of relevant information about the fundamental elements of recent electrochemical immunosensors developed against AD biomarkers (Figure 6a–c). Figure 6a provides a representation of used electrodes in recent research. It can be observed that the majority of studies targeted GEs interfaces (20%) in the development of related immunosensing platforms, which deliver excellent conductivity, high electron transfer rate leading to amplified electrochemical signals, ease of surface modification, high stability, and even the desired biocompatibility. GCE was the second most commonly used electrode (9%) due to its low cost, compatibility with various nanomaterials and biomolecules, and ease of customizing and integration with most immunosensor assemblies. The selection of components for immunosensors, especially electrodes, is crucial and depends on their compatibility with desired surface modifications. Gold is a highly versatile material for surface modification and is widely used due to its unique chemical and physical properties. The surface of the GE can be modified with various chemical groups, such as thiol, amine, and carboxylic acid, to facilitate the attachment of biomolecules such as antibodies or antigens. For example, thiol groups can form strong covalent bonds with gold surfaces by forming Au-S bonds. These functional groups allow immobilizing thiol-containing biomolecules such as antibodies or other biomolecules onto the GE surface. Similarly, carboxyl groups on the surface of an electrode can be used to attach amine-containing molecules, such as proteins or peptides, through amide bond formation. The ease of modifying GEs with different chemical groups makes them a popular choice for developing these biosensors The screen-printed electrodes (SPEs) are another type of used electrode (4%) in recent immunosensors due to their low-cost, disposability, and ease of use and modification for specific applications.

In addition, integrating nanostructures to modify the electrode surface has improved immunosensors with enhanced sensitivity, selectivity, and stability. They can increase the available surface area for biomolecule immobilization, which enhances the sensitivity of the immunosensor, improve electron transfer kinetics by providing a conductive pathway between the electrode and the biomolecules, and can enhance the stability of the immobilized biomolecules by preventing denaturation or degradation. For example, CNTs have been used to modify GEs in immunosensors due to their high surface area, excellent conductivity, and biocompatibility. CNTs can be functionalized with different chemical groups to facilitate the covalent or non-covalent attachment of biomolecules such as antibodies or antigens. The resulting CNT-modified electrodes have improved sensitivity and selectivity for detecting target analytes.

Commonly, antibodies are widely used in electrochemical immunosensors due to their high specificity and affinity for target analytes. Antibodies can be immobilized on the electrode surface using selective surface chemistry to capture and detect biomarkers. However, some drawbacks of using antibodies in electrochemical immunosensors include their high cost compared to other biomolecules, such as enzymes or DNA probes, and their sensitivity to environmental conditions such as pH and temperature, which may change their stability and performance. Therefore, researchers have explored using alternative biomolecules, such as aptamers, peptides, and molecularly imprinted polymers, to design electrochemical biosensors.

The early diagnosis of AD is crucial for the effective treatment and management of the disease. Figure 6b illustrates that the Aβ peptide is the most frequently analyzed analyte (~28%) in recent research. This finding suggests that Aβ is considered an important biomarker for the early diagnosis of AD due to its high selectivity and its role in the formation of plaque in brain cells. Overall, Aβ subtypes such as Aβ_(1-40)_ and Aβ_(1-42)_, as well as other protein biomarkers including t-tau, c-tau, p-tau_181_, p-tau_231_, p-tau_381_, and p-tau_441_ have been used for diagnosing AD in CSF and blood samples.

Electrochemical techniques such as voltammetry, impedance spectroscopy, and amperometry have greatly interested researchers for the purpose of detecting AD biomarkers. Figure 6c shows that DPV is the most commonly used electrochemical technique (16%) for detecting AD biomarkers mainly due to its high sensitivity. This technique is particularly useful for detecting low concentrations of analytes in complex biological matrices such as blood or CSF. In recent studies, EIS is another commonly used electrochemical detection technique (10%) for AD. EIS is particularly useful for detecting changes in interfacial properties, a promising technique for the electrochemical characterization of immunosensors for the early diagnosis of AD.

## 5. Challenges, Limitations, and Aspects for the Development of Electrochemical Immunosensors for AD

The challenges and limitations are essential for advancing electrochemical immunosensors and maximizing their potential in POCT. The development of efficient electrochemical immunosensors represents a highly promising approach to resolving the challenges associated with other detection techniques for diagnosing AD. These immunosensors offer a range of advantages, including rapid detection, good sensitivity, affordability, and portability, making them an ideal choice for POCT [96,112,113]. However, there is still a need for user-friendly platforms that not only provide ease of use but also deliver enhanced sensitivity and specificity for AD diagnosis. In addition, the simultaneous detection of multiple analytes is a critical issue in developing electrochemical immunosensors [114]. This challenge is particularly significant in detecting multiple AD biomarkers essential in accurate disease diagnosis and prognosis. Clinical applications prefer the simultaneous detection of several clinically relevant biomarkers, as relying on detecting a single biomarker is often inadequate. The simultaneous detection approach improves the diagnostic value and enhances the diagnostic process’s overall effectiveness [115,116].

There are several other challenges and limitations associated with electrochemical immunosensors. They may face limitations in terms of sensitivity, especially when detecting low concentrations of analytes or using complex biological samples [117,118]. Biological samples often contain various interfering substances that can affect the accuracy and reliability of electrochemical immunosensors. Long-term stability and reaching the desired reproducibility are other challenges due to factors such as electrode fouling, degradation of bioreceptors, and variations in assembly processes [96,119]. Developing cost-effective, miniaturized, and integrated electrochemical immunosensors while maintaining performance is a critical step to making them portable and user-friendly in POCT. Lastly, the lack of standardized methods and clinical validation of these immunosensors can cause a delay in their validation for application in clinics.

Selectivity is a very important aspect of developing an immunosensor for clinical settings. It allows for specific recognition and binding to the target analyte, even in interfering substances, ensuring accurate quantification and reliable detection. There are various reasons to lose the selectivity of immunosensors, including non-specific binding, cross-reactivity, and environmental factors (mainly temperature, pH, and humidity) [120]. Non-specific binding is considered one of the most significant factors affecting the selectivity of immunosensors. Non-specific binding can occur between non-target molecules present in biological samples (i.e., whole blood, plasma, and serum.) and the capture Ab, causing unreliable signals and reducing the sensitivity and selectivity of the immunosensor [121]. Non-specific binding can also lead to cross-reactivity, where the Ab interacts with other biomolecules (such as structurally similar molecules or analytes with similar binding sites). This type of binding can lead to false-positive signals where the immunosensor detects and responds to substances other than the target analyte, leading to the imprecise diagnosis of AD.

To minimize the effect of interference in immunosensing, various strategies can be employed, including the use of blocking agents to prevent non-specific binding, optimization of assay conditions to reduce cross-reactivity, using an appropriate binding matrix, processing of biological sample preparation (i.e., extraction, filtration, dilution, and so on), selection of the specific bioreceptors with the optimum affinity and immobilization method can help to reduce the interference effects, resulting in improving the selectivity of immunosensor.

The immobilization methods to attach the specific antibodies onto the electrode surface are important to determine the performance, especially the selectivity and stability of the immunosensor [122,123]. Covalent binding is one of the most effective immobilization strategies to improve the selectivity and stability of immunosensors, creating interactions between the surface of the signal interface and the functional groups of the bioreceptor (i.e., antibody) [124,125]. This method offers several advantages over other immobilization methods, including enhanced stability of the immunosensor via covalent binding attachment of the antibody to the electrode surface. This stability ensures that the antibody remains attached to the electrode surface under complex conditions, such as high pH, high temperature, high ionic strength media, and during several washing steps. Stability is also crucial for the long-term performance and reliability of the immunosensor. This strategy allows for the proper orientation of the bioreceptors on the signal interface, resulting in the binding sites of the bioreceptor being optimally positioned for interaction with target molecules by maximizing the specific binding between the bioreceptors and the target analyte [126,127]. In addition, covalent binding can also reduce non-specific binding by minimizing the exposure of the immunosensor to the sample matrix and improving the specificity and accuracy of the immunosensor. Covalent binding is a versatile method for immobilizing a wide range of antibodies, including monoclonal, polyclonal, fragment (e.g., an antigen-binding region (Fab)), and engineered antibodies (single-chain variable fragments (scFv)) [126,128,129]. This compatibility makes triggering a preferred method for developing highly sensitive and selective electrochemical immunosensors possible. For example, several chemical groups, such as thiol, amine, and carboxylic acid, can be used to treat the electrode surface, which facilitates the attachment of antibodies via covalent binding. Thiol groups, for instance, can create robust covalent bonds with gold surfaces by forming Au-S bonds. Furthermore, aminopropyltriethoxysilane/glutaraldehyde (APTES/GA) is widely used as a surface chemistry agent for the immobilization of antibodies on electrode surfaces via covalent binding. Using EDC/NHS chemistry is another possibility to activate the functional groups for covalent binding between the electrode surface and antibodies.

## 6. Conclusions and Future Perspectives

AD is a degenerative brain disorder that affects millions of people worldwide. It is a progressive disease that impairs cognitive abilities, memory, and physical function, making it challenging for individuals to perform daily tasks. Early diagnosis of AD is essential because it allows for timely intervention and management of the disease and can significantly improve the patient’s quality of life. However, accurate diagnosis can be complicated due to the need for early and reliable diagnostic techniques. Therefore, there is an urgent need to develop proper diagnostic techniques that are simple, rapid, reliable, cost-effective, and highly sensitive for the early detection and management of AD, which can ultimately improve AD patient outcomes.

Moreover, detecting blood-based biomarkers instead of CSF-based biomarkers is a critical component of the diagnostic protocol for AD. This approach can potentially increase accessibility to AD diagnosis for all patients, regardless of whether they exhibit AD symptoms. Blood-based biomarkers offer several advantages over CSF-based ones, including less invasive sampling procedures, easier accessibility, and low-cost detections. This review provided an overview of recent advancements in electrochemical immunosensors for detecting high-potential AD biomarkers (Aβ and p-tau protein) and their subtypes (AβO, Aβ_(1-40)_, Aβ_(1-42)_, t-tau, c-tau, p-tau_181_, p-tau_231_, p-tau_381_, and p-tau_441_) in biofluids, including blood and CSF. 

Various diagnostic methods, including electrochemical, electrical, mechanical, and optical techniques, have been investigated over time for AD. These methods aim to identify specific biomarkers associated with AD to facilitate early diagnosis and management of the disease. However, each method has its advantages and limitations. Electrochemical biosensors have shown great potential as a future diagnostic tool for AD due to their high sensitivity, simple production process, easy handling, portability, and low cost. Despite this potential, integrating these methods into clinical practice and diagnostic protocols is necessary to ensure their widespread use and effectiveness in AD management. Therefore, further research is needed to optimize electrochemical biosensors’ performance and establish their role in AD diagnosis. This review presented a comprehensive summary of the essential features and electrochemical performance of developed immunosensing platforms. The information encompasses electrode materials, nanomaterials or other signal amplifiers, biofunctionalization methods, and primary electrochemical sensing performance parameters, such as sensitivity, linear detection range, LOD, and clinical application. To sustain the growth momentum to develop diagnostic devices, it is necessary to overcome the challenges that must be addressed.

Integrating analytical possibilities on a single platform is crucial for developing advanced technologies. In this regard, developing electrochemical biosensors that enable the simultaneous detection of multiple biomarkers associated with AD can significantly enhance detection sensitivity while reducing false-positive/false-negative results. Detection can be improved by utilizing biorecognition molecules with different specificities equipped with suitable redox-active labels, allowing for the simultaneous detection of different AD biomarkers. However, mutual interference or overlapping of the electrochemical signals remains a significant challenge that might be addressed by identifying appropriate redox-active labels.

Another critical challenge in developing biosensors is improving their performance to a degree higher than physiological levels of biomarkers found in biological fluids while ensuring affordability and accessibility for point-of-care testing. In this regard, nanomaterials have emerged as promising candidates for enhancing biosensing sensitivity, particularly for early diagnosis and point-of-care applications. The application of nanomaterials and advancements in nanofabrication technologies and biomimetic surfaces can be further explored to develop biosensors that can detect AD biomarkers with even greater figures of merit.

## Figures and Tables

**Table 1:** Comparison of the assembly and analytical performances of electrochemical immunosensors developed for detecting Aβ peptides.

Analytes	Signal Transducer	Nanomaterials	Bioreceptors	Detection Techniques	Signal Marker	Real Sample	Detection Range	LOD	Ref.
Aβ	GE	AuNPs	Ab	EIS	[Fe(CN)_6_]^3−/4−^	Tear/Blood	1 pg mL^−1^–1 ng mL^−1^	1 pg mL^−1^	[71]
Aβ	Interdigitated microelectrode	- *	Ab	EIS	[Fe(CN)_6_]^3−/4−^	- ♣	0.1–100 pg mL^−1^	0.1 pg mL^−1^	[80]
Aβ	GCE	AuNPs-graphitic carbon nitride nanosheets (g-C_3_N_4_) nanocomposite/PdNPs	Ab	Electrochemiluminescence (ECL)	PdNPs@MOFs/K_2_S_2_O_8_	Serum	10 fg mL^−1^–50 ng mL^−1^	3.4 fg mL^−1^	[81]
Aβ	GE	AuNPs	Ab	Linear square voltammetry (LSV)	[Fe(CN)_6_]^3−/4−^	CSF	1 pM–50 nM	0.2 pM	[82]
Aβ	GE	Gold nanodendrites	Ab	Square-wave voltammetry (SWV)	Ru(NH_3_)_6_^3+^	Serum	10^−10^–10^−7^ mg mL^−1^	8.6 × 10^−12^ mg mL^−1^	[83]
Aβ	Gold nanopillar array	Nanopillar array	Ab	SWV	HRP/[Fe(CN)_6_]^3−/4−^	Tear	0.1–1 ng mL^−1^	0.14 ng mL^−1^	[84]
Aβ	GCE	CuSNPs@COFs nanocomposite/AuNPs	Aptamer/Ab	DPV	Thi-AuNPs/[Fe(CN)_6_]^3−/4−^	CSF	1 pM–1 μM	0.4 pM	[77]
Aβ_(1-40)_	Pt/Ir microelectrodes	-	Ab	EIS	- ♠	Brain tissue lysate samples	1–10^4^ pg mL^−1^	4.81 pg mL^−1^	[85]
Aβ_(1-42)_	GE	ZnO nanoarrays	Ab	DPV	[Fe(CN)_6_]^3−/4−^	Plasma	0.5–100 pg mL^−1^	62.3 fg mL^−1^	[68]
Aβ_(1-42)_	GE	Tantalum (Ta) nanolayer	Ab	DPV	[Fe(CN)_6_]^3−/4−^	-	2.2 pM–22 μM	1.62 pM	[75]
Aβ_(1-42)_	GCE	Highly ordered pyrolytic graphite nanoarray	Ab	DPV	FCA	-	10–200 ng mL^−1^	10 ng mL^−1^	[74]
Aβ_(1-42)_	SPGE	-	Ab	DPV	[Fe(CN)_6_]^3−/4−^	Plasma	1–1000 pg mL^−1^	1.4 pg mL^−1^	[70]
Aβ_(1-42)_	GCE	AuNPs	Ab	CV	FcOH/H_2_O_2_	Serum/Saliva	Serum: 0.056–13.7 nM/ Saliva: 0.056–41.2 nM	Serum: 25.2 pM/ Saliva: 23.8 pM	[76]
Aβ_(1-42)_	GCE	SnO_2_ nanofibers	Ab	EIS	[Fe(CN)_6_]^3−/4−^	Plasma	1 fg mL^−1^–1 ng mL^−1^	0.638 fg mL^−1^	[78]
Aβ_(1-42)_	3D nanostructure PC substrate	AuNPs	Ab	EIS	[Fe(CN)_6_]^3−/4−^	Plasma	10 pg mL^−1^–100 ng mL^−1^	113 fg mL^−1^	[73]
Aβ_(1-42)_	Glass/Ti/Au Interdigitated chain-shaped electrode	Gold nanoarray	Ab	EIS	[Fe(CN)_6_]^3–/4–^	Serum/CSF	10^−3^–10^3^ ng mL^−1^	100 pg mL^−1^ (serum)/ ∼500 pg mL^−1^ (CSF)	[86]
Aβ_(1-42)_	GCE	Fe_3_O_4_@polypyrrole-AuNPs nanocomposite/Flower-like Co-MOFs nanocomposite	Ab	ECL	Cobalt-based MOFs/*N*-(aminobutyl)-*N*-(ethylisoluminol) (ABEI)/[Fe(CN)_6_]^3–/4–^	Serum	10 fg mL^−1^–100 ng mL^−1^	3 fg mL^−1^	[87]
Aβ_(1-42)_	SPGE	Reduced graphene oxide (rGO)	Ab	DPV	[Fe(CN)_6_]^3−/4−^	Plasma	11 pM–55 nM	2.398 pM	[88]
Aβ_(1-42)_	GE	-	Ab	Capacitive	[Fe(CN)_6_]^3−/4−^	Serum	10–10^4^ pg mL^−1^	7.5 pg mL^−1^	[89]
Aβ_(1-40)_/Aβ_(1-42)_	GE	Conductive silk fibroin 3D nanostructure	Ab	LSV	Methionine (35)	Serum	2 pM–5 nM	Aβ_(1-40)_: 6.63 pg mL^−1^/ Aβ_(1-42)_: 3.74 pg mL^−1^	[90]
Aβ_(1-40)_/Aβ_(1-42)_	Au wire	Gold nanoarray	Ab	Chronoamperometry (CA)/DPV	[Fe(CN)_6_]^3−/4−^	Serum	0.1–1000 pg mL^−1^	Aβ_(1-40)_: 0.125 pg mL^−1^/Aβ_(1-42)_: 0.089 pg mL^−1^	[79]
Aβ_(1-40)_/Aβ_(1-42)_	Superwettable microchips	Vertical graphene@AuNPs nanocomposite	Ab	DPV	[Fe(CN)_6_]^3−/4−^/Ferrocene	Serum	0.1 pg mL^−1^–10 ng mL^−1^	Aβ_(1-40)_: 0.064 pg mL^−1^/Aβ_(1-42)_: 0.012 pg mL^−1^	[91]
Aβ	Magnetic glass carbon electrode	Fe_3_O_4_ NPs/ Mesoporous carbon nanospheres/Gold nanorods	Ab/Aptamer	ECL	Ru(bpy)_3_^2+^/gold nanorods	Serum	1 × 10^−5^–100 ng mL^−1^	4.2 × 10^−6^ ng mL^−1^	[92]
Anti Aβ_(1-40)_ Ab	SPCE	-	Aβ_(1-40)_ antigen	CV	-	Serum/CSF	1 ng mL^−1^–10 μg mL^−1^	1 ng mL^−1^	[93]

* Application of nanomaterial (s) not reported; ♣ Application of real samples not reported; ♠ Redox marker not reported.

**Table 2:** Comparison of the assembly and analytical performances of electrochemical immunosensors developed for detecting tau proteins.

Analytes	Signal Transducer	Nanomaterials	Bioreceptors	Detection Techniques	Signal Marker	Real Sample	Detection Range	LOD	Ref.
p-tau_181_	Au wire	Gold nanoarray	Ab	CA/DPV	[Fe(CN)_6_]^3−/4−^	Serum	0.1–1000 pg mL^−1^	0.176 pg mL^−1^	[79]
p-tau_181_/t-tau	Superwettable microchips	Vertical graphene@AuNPs nanocomposite	Ab	DPV	[Fe(CN)_6_]^3−/4−^/Ferrocene	Serum	0.1 pg mL^−1^–10 ng mL^−1^	p-tau_181_: 0.041 pg mL^−1^/ t-tau: 0.039 pg mL^−1^	[91]
p-tau_441_	ITO	rGO-AuNPs nanocomposite	Ab	EIS	[Fe(CN)_6_]^3−/4−^	Serum/CSF	1–500 pg mL^−1^	0.091 pg mL^−1^	[106]
p-tau_441_	GE	MWCNTs-rGO nanocomposite	Ab	DPV	[Fe(CN)_6_]^3−/4−^	Serum	0.5–80 fM	0.46 fM	[107]
t-tau	GE	- *	Ab	DPV	[Ru(NH_3_)_6_]^2+/3+^, [Fe(CN)_6_]^3−/4−^	Serum	0.968–454 pM	0.968 pM	[108]
p-tau_441_	GE	-	Ab	EIS	[Fe(CN)_6_]^3−/4−^	- ♣	10–100 μg mL^−1^	10 μg mL^−1^	[109]
p-tau_441_	SPCE	-	Ab	CV	[Fe(CN)_6_]^3−/4−^	CSF	0.0064–0.8 ng mL^−1^	0.0053 ng mL^−1^	[103]
p-tau	GE	Flower-shaped TiO_2_ nanostructure	Ab	EIS	[Fe(CN)_6_]^3−/4−^	Serum	1–200 ng mL^−1^	1.774 pg mL^−1^	[31]
tau	GCE	MnS-GO-PANI nanocomposite/AuNPs@Fe_3_O_4_ nanocomposite	Ab	DPV	[Fe(CN)_6_]^3−/4−^	Plasma	1 × 10^−13^–1 × 10^−6^ M	1 × 10^−14^ M	[101]
p-tau	GE	-	Ab	DPV	[Fe(CN)_6_]^3−/4−^	CSF/ Serum	0.05–3000 pM	0.02 pM	[110]
p-tau_181_	SPCE	MWCNTs-PtNPs nanocomposite	Ab	SWV	[Fe(CN)_6_]^3−/4−^	Serum	8.6–1100 pg mL^−1^	0.24 pg mL^−1^	[102]
p-tau_231_	GE	Gold nanoarray	Ab	EIS	[Fe(CN)_6_]^3−/4−^	Serum	10^−4^–10^1^ ng mL^−1^	140 pg mL^−1^	[99]
tau	GCE	PDDA nanolayer	Ab	EIS	[Fe(CN)_6_]^3−/4−^	Serum	0.01–100 ng mL^−1^	1.31 pg mL^−1^	[100]
c-tau	GE	-	Ab	DPV	[Fe(CN)_6_]^3−/4−^	Serum	10 pg mL^−1^–100 ng mL^−1^	0.1 pg mL^−1^	[104]
p-tau_381_	GE	AuNPs	Ab	DPV	[Fe(CN)_6_]^3−/4−^	Serum	0.5–100 pM	0.42 pM	[111]
tau	GE	Gold nanodendrites	Ab	SWV	Ru(NH_3_)_6_^3+^	Serum	10^−10^–10^−7^ mg mL^−1^	5.91 × 10^−11^ mg mL^−1^	[83]
p-tau_381_	CE	AuNPs-MoSe_2_ NSs nanocomposite	Aptamer/Ab	PEC	[Fe(CN)_6_]^3−/4−^	Serum	0.5 fM–1 nM	0.3 fM	[105]

* Application of nanomaterial (s) not reported; ♣ Application of real samples not reported.

## Data Availability

Not applicable.
